# Barriers to the non-acceptance of cervical cancer screenings (pap smear test) in women of childbearing age in a rural area of Peru

**DOI:** 10.3332/ecancer.2019.901

**Published:** 2019-01-31

**Authors:** Augusto Felix Olaza-Maguiña, Yuliana Mercedes De la Cruz-Ramirez

**Affiliations:** Academic Department of Obstetrics, Universidad Nacional Santiago Antúnez de Mayolo, Huaraz 02000, Peru

**Keywords:** refusal to participate, early cancer detection, cervical neoplasm, pap smear test, Peru

## Abstract

**Introduction:**

According to recent statistics, there is a high percentage of refusal of cervical cancer screening using the Pap smear test by women in rural communities in developing countries such as Peru. There are few studies on the reasons to explain said refusal in this country. The objective of this study was to determine the barriers related to the non-acceptance of cervical cancer screening using the Pap smear test in women of childbearing age from a rural Peruvian community.

**Methods:**

Cross-sectional study, with a total of 892 women of childbearing age from the rural Peruvian community of Marián, Huaraz province, Ancash region, who did not accept screening for cervical cancer using the Pap smear test during their care at the Marián Health Centre. A questionnaire given from September 2017 to April 2018 was used. The information was processed with the statistics programme for social science 22.0 programme, using the Chi-square statistical test.

**Results:**

For 4–6 years, 52.5% of women refused cervical cancer screening using the Pap smear test. The barriers that showed a statistically significant relationship to the non-acceptance of cervical cancer screening using the Pap test were socio-demographic (age, marital status, education level, occupation and home income) and institutional barriers (counselling for cervical cancer screening, importance of the gender of health personnel administering the screening, history of mistreatment by health personnel, fear or embarrassment of the screening procedure and a delay in sending the results) (*p* < 0.05); while a history of sexually transmitted infections and a feeling of physical well-being in sexual and reproductive health were the only related reproductive barriers (*p* <0.05), this was not demonstrated with the age of first sexual activity, number of sexual partners, age of first pregnancy and total number of pregnancies (*p* > 0.05).

**Conclusions:**

Socio-demographic and institutional barriers are related to the non-acceptance of cervical cancer screening using the Pap test in women of childbearing age in the rural Peruvian community of Marián, with a lesser emphasis on reproductive barriers. With this in mind, we recommend undertaking broader studies in populations of different age groups, which should be oriented towards the design and application of preventive/promotional programmes by health institutions to promote the participation of community workers with the goal of aiding the identification and control of said barriers, reducing the refusal of cervical cancer screenings by women from rural areas.

## Introduction

Cervical cancer continues to be a major cause of death for women worldwide, demonstrated by the fact that one person dies from it every 2 minutes, deaths that in 90% of cases occur in middle- and low-income countries [1, 2], such as those in Latin America and the Caribbean, where mortality rates are three times higher than in North America, a trend that would cause a 45% increase in deaths by the year 2030 if it continues [3].

In Peru, cervical cancer is the most common cancer diagnosis in women, with 24.1% [4, 5], according to the current data from the National Institute of Statistics and Informatics in this country, in which one women died every 5 hours from this cause in 2017 [6].

On the other hand, if the screening is followed by the treatment of the identified precancerous lesions, it is a cost-effective strategy for cervical cancer prevention, where human papilloma virus (HPV) vaccination can help to prevent approximately 70% of cases of this type of cancer [3]. While this strategy has been included in the most recent standards published in 2017 by Peru’s Ministry of Health as the National Plan for the Prevention and Control of Cervical Cancer 2017–2021 [4], which highlights molecular HPV testing as the most responsive and effective screening method for accurately identifying women who have a high risk of developing cervical cancer [3], the screening has unfortunately not yet been implemented across Peru [4, 5], especially in remote areas such as the sierra region, where social, economic and cultural conditions are the evidence of the enormous inequalities that exist within cities such as Lima, Peru’s capital, whose development figures contrast with the reality observed in rural villages [7–10], where the Pap smear test is used as a primary form of cervical cancer screening [6], and they have not achieved a decrease in the number of women comparable to the registered figures in those developed countries [11], a fact that, as the World Health Organization emphasises, is due not only to the limitations of the Pap smear test but also to the existing organisational mechanisms of the healthcare systems and cultural and community aspects [12].

As a result of what has been shown here, it is apparent that the coverage of screening using the Pap smear test is low in medium and low-income countries (43% on average) [13] compared to developed countries such as the USA (77.7%) [14], as well as that this situation affects women in rural and poor areas with greater emphasis [15], and we can observe that in Peru’s case, there is a difference in the performance of the test between urban and rural areas (64.5% and 56%, respectively) [6]. Likewise, according to recent statistics registered in developing countries, there is a high percentage of women in rural communities refusing cervical cancer screenings using the Pap test, with figures up to 71% in India [16], 67% in Colombia [17] and 84% in Brazil [18], and there are few studies [19–23] on the reasons behind this refusal by women from the rural areas of Peru, a geographic reality that pertains to the rural community of Marián, which was chosen for having the second highest cervical cancer screening refusal rate in the entire north sierra of Peru (53%), and the fact that there has not been a similar study undertaken in this geographic area [6].

Due to the considerations mentioned in the above paragraphs, the general objective of this research was to determine the barriers related to non-acceptance of cervical cancer screening using the Pap smear test in women of childbearing age from the rural community of Marián; the following specific objectives were established for this: identifying the socio-demographic barriers related to the non-acceptance of cervical cancer screening, determining the relationship of institutional barriers to the screening’s non-acceptance and identifying the reproductive barriers and their relationship to non-acceptance.

In that regard, this article reports the results with respect to the aforementioned objectives, as well as providing an analysis on the importance of performing actions intended to reduce the refusal of cervical cancer screenings by women from rural areas.

## Methods

A cross-sectional study was conducted. The population was made up of 892 women of childbearing age who met the following inclusion criteria: between 18 and 49 years old, no history of cervical cancer, no current pregnancy and the non-acceptance of cervical cancer testing using the Pap smear test, a refusal that occurred during their care in the Marián Health Centre, according to information recorded during the last 10 years as part of the Community Monitoring System (SIVICO) [24]. We considered the age from 18 to 49 because according to the norms and directives applied by Peruvian health establishments before 2017 [25, 26], this was the target age group for the Pap smear test, so women over 49 who refused to take the screening were not registered in the SIVICO, which limited the inclusion of these women in the present study. Likewise, we decided to work with the whole population, and for this purpose, we established a strategy of conducting home visits, allowing us to locate all of the women.

Taking into account the conceptual framework released by the Pan American Health Organization [27, 28] on the types of barriers that limit people’s participation in health institutions, we developed a questionnaire to obtain information, which was made up of four parts totaling 17 items: socio-demographic barriers (five items: age, marital status, education level, occupation and home income), institutional barriers (five items: counselling on cervical cancer screening, importance of gender of the health personnel administering the screening, history of mistreatment by health personnel, fear and/or embarrassment of the screening procedure and delay in sending the results), reproductive barriers (six items: age of first sexual activity, number of sexual partners, age of first pregnancy, total number of pregnancies, history of sexually transmitted infections (STIs) and feeling of physical well-being in sexual and reproductive health) and a non-acceptance period for cervical cancer screening (one item: number of years without accepting said screening). For the purpose of avoiding information or memory biases, the data compiled were compared against the information registered over the past 10 years in the Marián Health Centre’s SIVICO, with house visits being made again in cases where there were incongruencies or questions. The validity of the instrument was evaluated by means of expert opinions, the Kendall tau test of which showed a significance level of 0.001. Reliability was measured through Cronbach’s alpha, which was 0.841. The instrument was applied between September 2017 and April 2018 by the authors of the research and two other duly-trained people, understanding the requirement to include the prior voluntary signing of the informed consent declaration, and respecting data privacy throughout. The research protocol was presented in a timely manner to the Ethics Committee of the Marián Health Centre, which authorised its implementation.

The Statistics Program for Social Science 22.0 was used for information processing, employing the frequency tables and graphics as well as the Chi-square test as a tool for analysis, with a significance level of 0.05.

## Results

The total number of women considered in the study (892) completed the questionnaire and ranged from 18 to 49 years of age (mean = 34.7). The demographic characteristics of the study participants are provided in Table 1.

In Table 2, one can see that over 4–6 years, the largest percentage of women had declined cervical cancer screenings through the Papanicolaou test (52.5%), the majority (26.2%) of them being between 28 and 37 years of age, single (27.4%), with a primary school level of education (24.6%) and living as homemakers (18.2%) with an economic income below the living minimum wage set for Peru at S/. 930,000 Soles (279.86 USD) monthly (29.6%), demonstrating that all of the socio-demographic barriers studied displayed a statistically significant relationship with the non-acceptance of cervical cancer screenings through Papanicolaou testing (*p* < 0.05).

With regards to institutional barriers (Table 3), it can be seen that the majority of women with a period of non-acceptance of cervical cancer screening of between 4 and 6 years (30.3%) had not received consultation regarding said screening, said that the sex of the healthcare personnel member responsible for tending to them was an important factor (50.2%), had incident(s) of maltreatment on the part of said personnel members (38.9%), felt apprehension, fear and/or embarrassment about the screening procedure (41.5%), and experienced a delay in the delivery of the results (32.4%); leading to the finding of the statistically significant relationship between the aforementioned institutional barriers and the non-acceptance of cervical cancer screenings through the Papanicolaou test (*p* <0.05).

Likewise, as is shown in Table 4 regarding reproductive barriers, the largest percentage of women who had declined cervical cancer screening for up to 6 years had started having sexual relations at 18 years of age (34.4%), had a maximum of two sexual partners (28.7%) and two pregnancies in their lives (27.5%), which came about after 19 years of age (28.7%), had no history of STIs (33.1%), and felt that their sexual and reproductive health was in a good state (39.3%), demonstrating that, aside from the last two items (*p* <0.05), the rest of the reproductive barriers did not present a statistically significant relationship with the non-acceptance of cervical cancer screenings using the Papanicolaou test (*p* >0.05).

## Discussion

This research project found that the largest percentage of women in the Peruvian rural community of Marián had, over 4–6 years, declined to have a cervical cancer screening through a Papanicolaou test (52.5%), which is in contrast with the 79.5% of women in rural areas of Kenya [29] and 82.6% of women of various ethnicities in the United States of America who are inclined to submit to said screening, where 35.6% even prefer to have one done annually [14]. Nonetheless, what has been shown in the Peruvian community of Marián has a relation to the percentages of non-acceptance found in other studies carried out on rural populations of India (71%) [16], Central and South America (67% in Colombia [17] and 84% in Brazil [18]), in which cases the non-acceptance of the screenings by means of the Papanicolaou test was much greater, probably due to the fact that in these research cases, the lack of knowledge regarding the utility and benefits of the Papanicolaou test was considered an associated factor.

Studies carried out in Peru have emphasised the identification of barriers associated with non-acceptance of the Papanicolaou test, and have been carried out in urban populations, primarily in the biggest cities of the coastal region. Few studies have been done on rural communities, exceptions being the study by Bustamante in Cajamarca [20], Ramírez in Huánuco [19], Marticorena in Junín [21] and Cangalaya and Quispe in Huancavelica [23], which showed non-acceptance rates for cervical cancer screening of 37%, 38%, 57.7% and 61%, respectively, with the latter two percentages being notably closer to the figures reported in this research study (52.5%). Nonetheless, it is important to clarify that the aforementioned studies have been done on rural populations in southern Peru, where the linguistic and cultural reality is different to that of the geographic area corresponding to the northern sierra of said country, where the women of the Peruvian rural community of Marián live, a place where no study like this one had ever been done.

With regards to the socio-demographic barriers, the low coverage of the Papanicolaou test amongst the women in the rural area of Appalachia located in Virginia, United States of America, was related to low levels of income, deficient education levels and working from home (29%) [30], which were also the reasons mentioned in the study by Austad *et al* [31] on the rural populations of Guatemala, where scarce financial income ended up being crucial for 20% of the women. Likewise, two research studies done in Colombia reported the existence of socio-demographic barriers impeding the timely diagnosis of cervical cancer by means of the Papanicolaou test, such as age, civil status, education level and financial income [17, 32], all of which share not only a similarity to the findings of this investigation, in which said barriers were also associated, but also with the study by Ramírez on the Peruvian locality of Huánuco [19]. In addition, another research study developed by Barrionuevo with women of diverse rural areas in Peru also concluded that low education levels and scarce economic resources were the barriers of exclusion and inequality to cervical cancer screenings [22].

With regard to the results of this research as to institutional barriers, the study by Maza *et al* [33] carried out on women in rural areas of El Salvador found that, amongst the most commonly-cited reasons for not accepting assistance with the Papanicolaou test was that they felt embarrassed being examined by a doctor of the male sex (55.6%), lacked trust in the healthcare personnel (9.5%), believed that the screening would be painful (27.1%), had an aversion to pelvic examination (19.9%) and long wait times (22.5%), barriers that, with a few discrepancies, were also evaluated in this present study with percentages greater than 58.6%. Meanwhile, other reasons cited in the study by Maza *et al* [33] were not considered in the research, such as fear of needing treatment (20.5%) and they believe that the results of the test would not be kept confidential (20.1%), as these were aspects left for consideration in future studies.

A study on rural populations in another Central American country like Guatemala showed that fears related to the Papanicolaou test procedure were also referenced by the women of said country (14.8%), as well as waiting periods for the delivery of the results (9%) [31], whereas in the case of Chile, where the healthcare system is much more organised than in the rest of South America, including in Peru, the reasons relating to fear, embarrassment, discomfort or maltreatment on the part of healthcare personnel were obstacles to having the Papanicolaou test done [34, 35], maltreatment that was similarly referenced by women in rural populations of Colombia [17, 32], with this situation also being mentioned by 71.5% of the women in this study, constituting a worrying actuality that merits further research and, if this is the case, immediate corrective actions on the part of the authorities.

Another study on a more distant population such as the one by Senthil *et al* [16] in India also shows similarities with regard to the fears referenced by the women regarding the screening procedure, as well as the lack of consultation on the part of healthcare personnel regarding detection and treatment of cervical cancer, a failing that, in the case of the women in the rural community of Marián, was given 53.2% of the time, whereas in the rural Appalachian region of the United States, discomfort with male providers and low levels of confidence in the doctors and healthcare system (30%) made up the primary reasons given [30].

According to the above, the contribution of this study to the current body of knowledge is fundamentally centred on the findings relating to the existence of institutional barriers when it comes to the non-acceptance of cervical cancer screenings by women of childbearing age living in rural regions such as the Peruvian community of Marián (Table 3), especially with regards to maltreatment perpetrated by healthcare personnel and the absence of consultations, issues that have not been addressed in other studies focussing on the identification of barriers associated with the non-acceptance of Papanicolaou tests in Peru’s rural populations [19–23]. New studies should be done to allow for improvements to be made in the work policies currently employed by this country’s Ministry of Health.

With regard to reproductive barriers, the study by Maza *et al* [33] in El Salvador concluded that the barriers involved in the decision not to accept the Papanicolaou test were an absence of symptoms (38.9%) and the belief that the test was unnecessary (27.5%), findings also shared with Senthil *et al* [16] in India, where the subjective feeling of well-being constituted a significant reason; it bears mentioning that the other reproductive characteristics such as age when sexual relationships begin, the number of sexual partners, the age at first pregnancy, total number of pregnancies and STI background were addressed in some studies as a risk factor for cervical cancer development [17, 19], but not as barriers for non-acceptance of screenings through Papanicolaou tests, and there was no significant result in this study.

As concluded in the studies by Murillo *et al* [36], Sierra *et al* [37] and Forman and Sierra [38], Peru, along with the rest of the countries in Central and South America, has implemented a cervical cancer screening programme that has not had the impact that was hoped for it with regards to mortality rates over the years, likely due to there not being any emphasis on the pluri-cultural reality of the population, particularly the communities with fewer resources and other forms of limitations [39], requiring not only the effective implementation of new screening alternatives, but also the application of preventive/promotional strategies on the part of duly-trained professionals in conjunction with health-care agents in the community.

On the other hand, it is important to acknowledge the limitations that have arisen throughout the completion of this study, such as the lack of information in the registries of the Marián Health Centre relating to the women older than 49 years of age declining to undergo the Papanicolaou test, a segment of the population that, through the changes established by Peru’s Ministry of Health beginning in 2017 and in light of the findings reported in this article, should be included in subsequent studies. Furthermore, the fluent use of Quechua by the survey personnel could have caused a bias during the compilation of the information, adding to the lack of previous research in rural populations in the same geographic area.

## Conclusion

The non-acceptance of cervical cancer screening in women of reproductive age in the Peruvian rural community of Marián is related to socio-demographic and institutional barriers, while a smaller relation is found to reproductive barriers.

It is recommended that comprehensive epidemiological studies be carried out on different age group populations in order to clarify how the barriers that were studied are related to the refusal of women from rural areas to undergo cervical cancer screening through Pap smear exams.

In a multilingual and multicultural country such as Peru, the application of preventative and promotional programmes based on the joint participation of family health professionals and community leaders can make it easier to identify and control various barriers in order to reduce the lack of acceptance of cervical cancer screening.

## Conflicts of interest

The authors declare that there are no conflicts of interest.

## Authors’ contributions

AFOM was in charge of designing the research protocol, collecting information and drafting the article; while YMDR looked for and revised bibliographic references, participating in information collection, statistical analysis and the drafting of the article.

All of the authors have reviewed and approved the final version of the article.

## Financial disclosure

This research work has been completely self-financed by the authors.

## Figures and Tables

**Table 1.** Demographic characteristics.

Characteristic	N°	%
Age	34.7 (range: 18–49)	
18–27 years	220	24.7
28–37 years	408	45.7
38–49 years	264	29.6
Marital status		
Single	413	46.3
Married	231	25.9
Cohabiting	215	24.1
Other	33	3.7
Educational level		
Illiterate	43	4.8
Primary school	429	48.1
Secondary school	309	34.7
Higher education	111	12.4

**Table 2.** Socio-demographic barriers related to the non-acceptance of cervical cancer screening.

Socio-demographic barriers	Non-acceptance period						Total		Results of Chi-square test
	1–3 years		4–6 years		≥ 7 years				
	**N°**	**%**	**N°**	**%**	**N°**	**%**	**N°**	**%**	
Age									
18–27 years	102	11.4	92	10.4	26	2.9	220	24.7	
28–37 years	115	12.9	234	26.2	59	6.6	408	45.7	X^2^ = 74.008
38–49 years	42	4.7	142	15.9	80	9	264	29.6	*p* = 0.000
Total	259	29	468	52.5	165	18.5	892	100	
Marital status									
Single	97	10.8	244	27.4	72	8.1	413	46.3	
Married	80	9	102	11.4	49	5.5	231	25.9	X^2^ = 24.232
Cohabiting	70	7.8	112	12.6	33	3.7	215	24.1	*p* = 0.001
Other	12	1.4	10	1.1	11	1.2	33	3.7	
Total	259	29	468	52.5	165	18.5	892	100	
Educational level									
Illiterate	6	0.7	17	1.9	20	2.2	43	4.8	
Primary school	122	13.7	219	24.6	88	9.8	429	48.1	X^2^ = 33,830
Secondary school	103	11.5	164	18.4	42	4.8	309	34.7	*p* = 0.000
Higher education	28	3.1	68	7.6	15	1.7	111	12.4	
Total	259	29	468	52.5	165	18.5	892	100	
Occupation									
Housewife	94	10.5	162	18.2	35	3.9	291	32.6	
Student	44	4.9	116	13	33	3.7	193	21.6	X^2^ = 62,408
Public and/or private sector employee	66	7.4	67	7.5	70	7.9	203	22.8	*p* = 0.000
Other	55	6.2	123	13.8	27	3	205	23	
Total	259	29	468	52.5	165	18.5	892	100	
Economic income at home									
< S/. 930.00 soles	179	20.1	264	29.6	86	9.6	529	59.3	X^2^ = 15.475
≥ S/. 930.00 soles	80	8.9	204	22.9	79	8.9	363	40.7	*p* = 0.000
Total	259	29	468	52.5	165	18.5	892	100	

**Table 3.** Institutional barriers related to the non-acceptance of cervical cancer screening.

Institutional barriers	Non-acceptance period						Total		Results of Chi-square test
	1–3 years		4–6 years		≥ 7 years				
	**N°**	**%**	**N°**	**%**	**N°**	**%**	**N°**	**%**	
Counselling about screening									
Yes	152	17	198	22.2	68	7.6	418	46.8	X^2^ = 20.558
No	107	12	270	30.3	97	10.9	474	53.2	*p* = 0.000
Total	259	29	468	52.5	165	18.5	892	100	
Importance of the gender of the health personnel administering the screening									
Yes	209	23.4	448	50.2	158	17.7	815	91.3	X^2^ = 52.710
No	50	5.6	20	2.3	7	0.8	77	8.7	*p* = 0.000
Total	259	29	468	52.5	165	18.5	892	100	
History of mistreatment by health personnel									
Yes	166	18.6	347	38.9	125	14	638	71.5	X^2^ = 10.054
No	93	10.4	121	13.6	40	4.5	254	28.5	*p* = 0.007
Total	259	29	468	52.5	165	18.5	892	100	
Apprehension, fear, and/or embarrassment due to screening process									
Yes	170	19	370	41.5	132	14.8	672	75.3	X^2^ = 18.537
No	89	10	98	11	33	3.7	220	24.7	*p* = 0.000
Total	259	29	468	52.5	165	18.5	892	100	
Delay in the delivery of screening results									
Yes	119	13.3	289	32.4	115	12.9	523	58.6	X^2^ = 27.393
No	140	15.7	179	20.1	50	5.6	369	41.4	*p* = 0.000
Total	259	29	468	52.5	165	18.5	892	100	

**Table 4.** Reproductive barriers related to the non-acceptance of cervical cancer screening.

Reproductive barriers	Non-acceptance period						Total		Results of Chi-square test
	1–3 years		4–6 years		≥ 7 years				
	**N°**	**%**	**N°**	**%**	**N°**	**%**	**N°**	**%**	
Age of beginning of sexual activity									
< 18	91	10.2	161	18.1	51	5.7	303	34	X^2^ = 0.885
≥ 18 years	168	18.8	307	34.4	114	12.8	589	66	*p* = 0.643
Total	259	29	468	52.5	165	18.5	892	100	
Number of sexual partners									
1-2	144	16.1	256	28.7	84	9.4	484	54.2	X^2^ = 0.970
≥ 3	115	12.9	212	23.8	81	9.1	408	45.8	*p* = 0.616
Total	259	29	468	52.5	165	18.5	892	100	
Age of first pregnancy									
< 19 years	46	5.1	82	9.2	37	4.2	165	18.5	
≥ 19 years	145	16.3	256	28.7	78	8.7	479	53.7	X^2^ = 3.895
Has never been pregnant	68	7.6	130	14.6	50	5.6	248	27.8	*p* = 0.420
Total	259	29	468	52.5	165	18.5	892	100	
Total number of pregnancies									
1–2	128	14.3	245	27.5	84	9.4	457	51.2	
≥ 3	63	7.1	93	10.4	31	3.5	187	21	X^2^ = 2.912
Has never been pregnant	68	7.6	130	14.6	50	5.6	248	27.8	*p* = 0.573
Total	259	29	468	52.5	165	18.5	892	100	
History of STIs									
Yes	148	16.6	173	19.4	59	6.6	380	42.6	X^2^ = 31.636
No	111	12.4	295	33.1	106	11.9	512	57.4	*p* = 0.000
Total	259	29	468	52.5	165	18.5	892	100	
Feeling of physical well-being									
Yes	150	16.8	350	39.3	128	14.3	628	70.4	X^2^ = 27.774
No	109	12.2	118	13.2	37	4.2	264	29.6	*p* = 0.000
Total	259	29	468	52.5	165	18.5	892	100	
